# Mass Spectrometry-Based Metabolomics Combined with Quantitative Analysis of the Microalgal Diatom (*Chaetoceros calcitrans*)

**DOI:** 10.3390/md18080403

**Published:** 2020-07-30

**Authors:** Awanis Azizan, M. Maulidiani, Rudiyanto R., Khozirah Shaari, Intan Safinar Ismail, Norio Nagao, Faridah Abas

**Affiliations:** 1 Laboratory of Natural Products, Institute of Bioscience, Universiti Putra Malaysia, Serdang 43400, Selangor, Malaysia; awanis_azizan@yahoo.com (A.A.); maulidiani@umt.edu.my (M.M.); khozirah@upm.edu.my (K.S.); safinar@upm.edu.my (I.S.I.); 2 Faculty of Science and Marine Environment, Universiti Malaysia Terengganu, Kuala Nerus 21030, Terengganu, Malaysia; 3 Faculty of Fisheries and Food Science, Universiti Malaysia Terengganu, Kuala Nerus 21030, Terengganu, Malaysia; rudiyanto@umt.edu.my; 4 Department of Chemistry, Faculty of Science, Universiti Putra Malaysia, Serdang 43400, Selangor, Malaysia; 5 Laboratory of Marine Biotechnology, Institute of Bioscience, Universiti Putra Malaysia, Serdang 43400, Selangor, Malaysia; norio.nagao@upm.edu.my; 6 Department of Food Science, Faculty of Food Science and Technology, Universiti Putra Malaysia, Serdang 43400, Selangor, Malaysia

**Keywords:** microalgal diatom, metabolic profiles, algalomics, chemical markers, UHPLC-MS

## Abstract

Although many metabolomics studies of higher land plant species have been conducted, similar studies of lower nonland plant species, which include microalgae, are still developing. The present study represents an attempt to characterize the metabolic profile of a microalgal diatom *Chaetoceros calcitrans*, by applying high-resolution mass spectrometry detection, via Q-ExactiveTM Plus Orbitrap mass spectrometry. The results showed that 54 metabolites of various classes were tentatively identified. Experimentally, the chloroform and acetone extracts were clearly distinguished from other solvent extracts in chemometric regression analysis using PLS, showing the differences in the *C. calcitrans* metabolome between the groups. In addition, specific metabolites were evaluated, which supported the finding of antioxidant and anti-inflammatory activities. This study also provides data on the quantitative analysis of four carotenoids based on the identification results. Therefore, these findings could serve as a reliable tool for identifying and quantifying the metabolome that could reflect the metabolic activities of *C. calcitrans*.

## 1. Introduction

**Figure 1:**
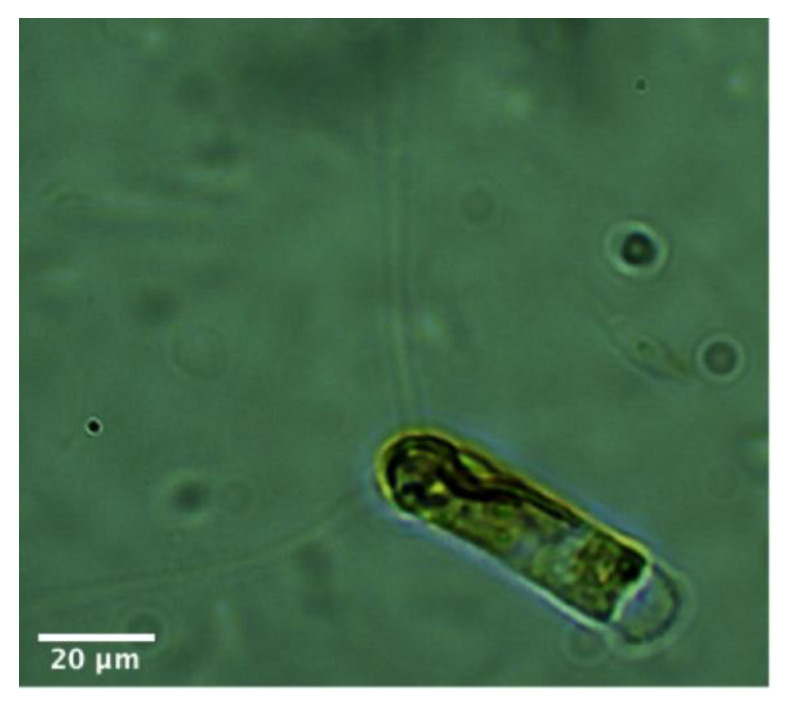
*Chaetoceros calcitrans*.

The marine diatom *Chaetoceros calcitrans*, as shown in Figure 1, is unialgal from the class Bacillariophyceae and has long been used in the mariculture industry. Existing research studies show that this microalga presents high metabolic diversity and that it contains materials that can potentially be used as a valuable source of food supplements for human consumption and as a source of therapeutic ingredients for curing human diseases and improving the survival of mariculture species [1,2]. Given their ability to produce photoprotective and photosynthetic pigments, fatty acids, lipids, polysaccharides, amino acids, and sugar–alcohol metabolites, the genus *Chaetoceros* is well appreciated for its anti-cancer, antioxidant, anti-inflammatory, and anti-tuberculosis activities [1,3,4,5]. Their bioactive metabolites can play important roles in future as chemotaxonomic markers for the identification of species or taxa and for the assessment of biodiversity in its marine habitats in the production of organic matter by phytoplankton microalgae, geographic variation, and seasonality in productivity, thereby revealing local and global changes in marine ecosystems [6]. However, it is also likely that their bioactive metabolites can be altered by changes in gene expression, regulation of protein functions, and in response to changes of different life stages and growth conditions [7]. Therefore, it is of interest to understand more about what is happening to the cellular systems of the diatom species *C. calcitrans* and to identify potential new molecules since they are ecologically and nutritionally significant to society in diverse ways.

The measurement of metabolites can be done using metabolomics and the application of this approaches in diatom research to investigate candidate biomarkers for human health purposes are developing rapidly. In comparison with other omics approaches, including genomics, transcriptomics, and proteomics, metabolomics is still considered as a new aspect in marine diatom research. Thus far, metabolomics-based approaches have proven useful for assessing the effects of Fe limitation in *Phaeodactylum tricornutum* which they have also combined with the transcriptomic data [8,9], the variations of metabolites in different culture conditions (heterotrophic and mixotrophic) in a green microalga food supplement ‘*Euglena*’ [10], key metabolite markers in different growth phases in *Skeletonema marinoi*, and metabolomics features under different growth phase-dependent markers in cultured diatoms *Chaetoceros teneuissimus* [7]. Most of these data have been acquired by a GC-MS technique due to its high sensitivity for the identification of volatile and thermally stable metabolites. Therefore, metabolomics offers a unique potential to unravel more on the metabolic features of *C. calcitrans* underlying the suitable extraction method used and how the identified features are affiliated to the bioactivities.

Previous findings obtained using 1D and 2D NMR spectroscopy via the metabolomics approach revealed that 29 metabolites belonging to various classes, including carotenoids, chlorophylls, fatty acids, amino and organic acids, cholesterol simple sugars, and sugar-alcohol metabolites, were tentatively identified [3]. In addition, the findings showed that there is an established correlation between the metabolite compositions with the biological activities of the microalga extracts prepared from different solvent extraction ranging from polar, semi-polar, and non-polar solvents. Nevertheless, a challenging issue that we are facing using the NMR technique is the congestion and overlapping signals. Hence, further examination of the health-promoting metabolites of *C. calcitrans* using other techniques is believed to be meaningful to overcome these shortcomings and exploring the potential pharmacological relevance of marine diatom.

To obtain a comprehensive ‘physiological snapshot’ of the diatom cells, advanced analytical tools such as ultra-high performance liquid chromatography–mass spectrometry (UPLC-MS) analysis has been used to assess the metabolic level in diatoms, since this tool can provide information on the possible presence of chemical compounds and differentiate the structural classes of metabolites present in minor or trace amounts, without requiring any derivatization across the inherent diversity of metabolites. In interesting works published by Bianco et al. [11] and Cataldi et al. [12] showing the usefulness of the high-resolution MS to determine the positional isomers, the authors proposed a strategy to use the combination of soft-ionization techniques with high-energy collisional dissociation (HCD). The application of such a strategy enabled the location of C=C bond between the carbon 7th and 8th of the acyl chain of *N*-Acylhomoserine lactones (AHLs) discovered in bacteria isolates when a standard LC method is ineffective to resolve the separation of compounds. Within this high-resolution MS/MS capability, the resolved peaks of isobaric precursors or fragments of several classes of metabolites including carotenoids, fatty acids, triacylglycerol, and diacylglycerol enabled accurate assignments to the peak intensity of the diagnostic ions. This is also commonly performed in combination with ultraviolet diode array detectors (UV-DAD). Considering the wide range of metabolites present in *C. calcitrans* and the influence of solvent extraction when polarity remains indispensable in metabolite extraction, the used of targeted UHPLC-MS in metabolomics analysis is considered to be of importance in revealing a more complete picture of the metabolome [13,14]. Nevertheless, no metabolite profiles data are currently available concerning the *C. calcitrans* using the UHPLC-MS approach. Therefore, this study is conducted to fill the gap in current research analysis in monitoring the whole set or at least the medicinally relevant metabolome with no deliberate bias as the UHPLC-MS offers higher sensitivity and selectivity, is quantitatively accurate, and requires smaller amounts of sample than other tools [6].

The present work aims to resolve individual metabolites (polar, semi-polar, and non-polar analytes) extracted from *C. calcitrans* into separate peaks, enhancing the opportunity to uncover additional metabolites using UHPLC-ESI-Orbitrap MS. High-resolution-MS detection using the Q-Exactive^TM^ Plus Orbitrap mass spectrometer, which is equipped with Thermo Scientific™ Xcalibur™ software that provides method setup, data acquisition, data processing, and reporting. This benchtop LC-MS system allows the differentiation of minor isotopes, offers greater mass resolutions, mass accuracy and faster scan speed for the analysis of low-abundance metabolites [15]. The tentatively identified metabolites that are found in positive and negative ion modes were listed and discussed. Multivariate data analysis (MVDA), both supervised and un-supervised, was used to classify and relate the identified chemical markers to the ability to protect against oxidation and inflammation. The quantitative analysis of the targeted metabolites of great interest was then assessed to reliably confirm their presence and amount.

## 2. Results and Discussion

### 2.1. Identification of Metabolites and Profiling of C. calcitrans Extract by UHPLC-ESI-Orbitrap MS/MS

**Figure 2:**
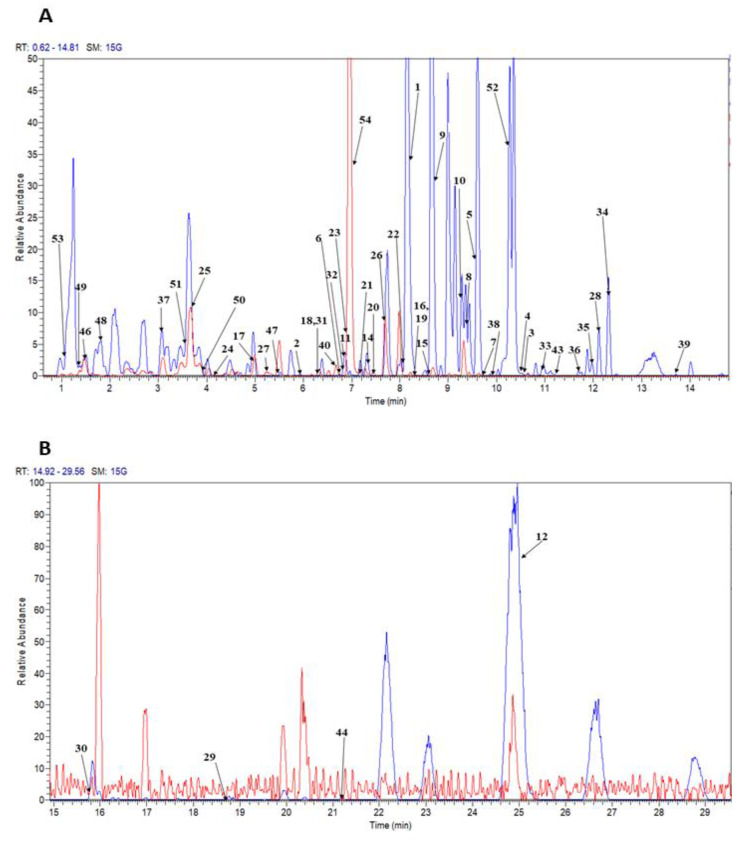
UHPLC-ESI-Orbitrap MS base peak chromatogram of the chloroform extract (**A**) Retention time 0–15 min (**B**) Retention time (15–30 min) obtain from microalga diatom *C. calcitrans*. Blue color represents the chromatogram acquired in positive ion mode whereas red color represents the negative ion mode. For peak assignment see Table 1.

The data variations resulting from the phytochemical screening analysis in our previous study [3] suggested that the type of metabolites presented in each extract is a primary factor. To obtain a convincing identification of the metabolites group presented in the extracts, UHPLC-ESI-Orbitrap MS analysis was performed. A total of 54 metabolites belonging to the classes of carotenoids, chlorophylls, fatty acids, glycerolipids, glycerolphospholipids, sterol lipid, (C20 isoprenoids) diterpenes, *N*-acyl-alpha amino acids, and their derivatives, benzoic acid esters, fatty acid esters, fatty amides, and fatty aldehydes, were tentatively characterized in crude extract of *C. calcitrans* prepared from chloroform (Figure 2) by matching the accurate masses, fragmentation pathways, and MS/MS spectral data with the database established and with the available analytical standards. By visual inspection of the representative chromatographic spectrum (Figure 2), the chloroform followed by acetone extracts possessed more intense and prominent peaks with some significant differences compared to the other extracts (hexane, methanol, and 70% ethanol). Therefore metabolite identification focused on both extracts in the present study. Each of the mass spectra belong to these extracts was provided in the Supplementary Materials for comparison (Supplementary Figures S1–S4). In addition, both extracts exhibited the most significant DPPH free radical scavenging and nitric oxide inhibitory activities [3]. When no analytical standards were available for most of the identified compounds, or if they are expensive, identification of the specific metabolites was based upon chromatographic and spectral information, such as retention times, UV spectra, and the presence of fragment ions as reported in the literature. Many of the targeted metabolites were processed using Mass Frontier 3.0 (Thermo Fisher Scientific, San José, CA, USA) to facilitate the prediction of the fragmented ions in both the protonated and deprotonated molecular ion modes using the general and library fragmentation mechanisms. A detailed analysis of each individual metabolite is tabulated in Table 1.

#### 2.1.1. Identification of Carotenoids and Chlorophylls

In the present study, a total of 13 natural pigments were identified in extracts of *Chaetoceros calcitrans;* of these, 10 were carotenoids (peaks 1–10) and peaks 11–13 represented chlorophyll and chlorophyll transformation products. Among the carotenoids identified, fucoxanthin was the main pigment accumulated by this marine diatom. Fucoxanthin (Peak 1) which has the empirical molecular formula C_42_H_58_O_6,_ is indicated in the MS/MS data as listed in Table 1, its UV-vis spectrum at (*t*_R_ = 8.19; λmax = 412, 442 nm) showed an [M + H]^+^ ion at *m*/*z* 659.4312 [16,17]. For fucoxanthin, the feature of product ions obtained from protonated molecular ions was observed at *m/z* 641.4180; corresponding to the loss of water and *m/z* 581.3969 resulted from the loss of C_2_H_9_O_3_. The deacetylated metabolite fucoxanthinol (Peak 2) found in the MS/MS spectrum produced the adduct ions [M + Na]^+^ (*m*/*z* 599.4075) and [M + H − H_2_O]^+^ (*m*/*z* 581.3972) [17].

Peaks 3 and 4 were assigned to lutein (*t*_R_ = 10.54) and zeaxanthin (*t*_R_ = 10.53), respectively. They showed protonated ions at *m*/*z* 569.4210 and 569.4204, respectively. The resolution of lutein and zeaxanthin remained inadequate, and this caused them to coelute as two very close chromatographic peaks. The mass error for both metabolites were high, we speculated that this is attributed to the complex mixtures of crude extracts, very low abundance of peaks 3 and 4 and improper calibration of the system. Nevertheless, the confirmation of identification from those metabolites was achieved based on their elution time and comparison with the analytical standards [18,19]. Both metabolites are isomers and display similar mass spectrometric behavior, yielded the fragment ion *m*/*z* 523.5612. The fragment ion was formed due to loss of C_2_H_5_O. In the MS^2^ spectrum of (3′,3′)-astaxanthin in peak 5, the protonated molecular ion at *m*/*z* 597.3903 was eluted at retention time (*t*_R_ = 9.56). However, the fragment ion *m*/*z* 147.1163, which corresponds to the dehydrated terminal ring with loss of water and cleavage at the 7,8 bond (C_10_H_11_O), was also found at quite an intense level [18].

Peak 6 was identified and characterized as the violaxanthin isomer (also known as neoxanthin); it corresponded to the loss of one [M + H − H_2_O]^+^ and two water [M + H − 2H_2_O]^+^ moieties. In the MS/MS experiments on neoxanthin, the protonated ion, *m*/*z* 601.4238 was present [6,18]. The fragment ions at *m*/*z* 583.4016 [M + H − H_2_O]^+^ and *m*/*z* 565.3912 [M + H − 2H_2_O]^+^ indicated a loss of water from the protonated ion. Peak 7, which was assigned as diadinoxanthin (*t*_R_ = 9.75), yielded the [M + H]^+^ ion at *m*/*z* 583.4135 in its MS/MS spectrum [6,18]. The presence of two signals already interpreted as diagnostic fragments of *m*/*z* 565.4039 [M + H − 2H_2_O]^+^ (generated by the loss of two water molecules) and *m*/*z* 547.3956 [M + H − H_2_O]^+^ (produced by the loss of one water molecule from *m*/*z* 565.4039 after further splitting) were observed.

In the MS/MS experiments, peak 8, which displayed the [M + H]^+^ ion at 581.3989 was identified as (3*S*,4*S*,3′*R*)-4-hydroxyalloxanthin [6]. The ion at *m*/*z* 563.3879 (C_40_H_52_O_3_) was formed by the loss of H_2_O, whereas the ion at *m*/*z* 489.3367 (C_40_H_52_O_3_) showed the loss of C_4_H_11_O_2_ from its precursor ion. Peaks 9 and 10 were determined as canthaxanthin (*t*_R_ = 8.76) at [M + H]^+^ (*m*/*z* 565.4026) and 14′-apo-beta-carotenal (*t*_R_ = 9.23) at [M + H]^+^ (*m*/*z* 311.2369) in their MS/MS positive spectra. In the MS/MS spectrum of canthaxanthin, the fragment ion at *m*/*z* 547.3898 (C_40_H_51_O) results from the loss of H_2_O. On the other hand, 14′-apo-beta-carotenal generated fragment ions of [M + H − C_2_H_4_]^+^, [M + H − C_2_H_4_O]^+^ and [M + H − C_3_H_4_O]^+^ at *m*/*z* 283.2412, 267.2106 and 255.2108, respectively, in its high resolution MS/MS spectrum, being great utility for structural confirmation and elucidation [18].

A fragment ion at *m*/*z* 119.09 was observed in lutein, zeaxanthin, violaxanthin, diadinoxanthin, (3*S*,4*S*,3′*R*)-4-hydroxyalloxanthin and 14′-apo-beta-carotenal. The known fragment ion at *m*/*z* 119.09 found by Fu, et al. [16] had been confirmed in this experiment, representing the cleavage between carbons 9,10 and 13′,14′.

The metabolite at peak 11 was tentatively identified as chlorophyll *c*_2_ and peaks 12 and 13 were found to contain chlorophyll transformation products. Peak 11 was identified as chlorophyll *c*_2_ (*t*_R_ = 6.88) based on the [M + H]^+^ (*m*/*z* 609.4522), revealing the loss of carbomethoxy group from the protonated molecular ion, signal at *m/z* 549.1774 and *m*/*z* 591.44, corresponding to the elimination of water [20]. Peaks 12 and 13 were identified as pheophytin *a* and pheophytin *b* as observed in the MS/MS spectra, which showed characteristic peaks at [M + H]^+^ (*m*/*z* 871.5739) and [M + H]^+^ (*m*/*z* 885.5518) with elution times of (*t*_R_ = 25.49) and (*t*_R_ = 19.26), respectively [20]. The fragment ions observed at *m*/*z* 593.2754 and *m*/*z* 593. 5330 were occurred due to the loss of the phytol group, C_20_H_38_. Collectively, the distribution of the identified cellular pigments suggested that fucoxanthin was more common in diatom *C. calcitrans*. The observed high peak intensity of all the detected peaks might be explained by the response of this photosynthetic pigment to white light condition and the experiments was best to be performed during transition of exponential and start of stationary phase [8,9]. This may also be attributed to the great sensitivity of the high-resolution mass spectrum employed here. The semi-polar solvent such as chloroform and acetone were more appropriate extraction solvents to explore the key-targeted metabolites in the diatom cells of *C. calcitrans*.

#### 2.1.2. Identification of Fatty Acids

Information obtained from negative ionization from MS/MS revealed the presence of characteristic peaks corresponding to fatty acids in *C. calcitrans* extracts; these are listed in Table 1. Thirteen metabolites with intense deprotonated molecular ions in either the MS or MS/MS spectrum were tentatively identified as fatty acids. The metabolite at peak 14 was assigned as myristic acid [M − H]^−^ (*m*/*z* 227.2104) (*t*_R_ = 7.31) [21]. No fragment ion that matched a reference in the online database or the literature generated from the precursor ion. Peak 15 was identified as palmitic acid [M − H]^−^ (*m*/*z* 255.2330) (*t*_R_ = 8.58) [22,23]. Its characteristic fragment was observed at *m*/*z* 61.9871 (C_2_H_5_O_2_) due to loss of C_14_H_25_ from the precursor ion. The metabolites at peaks 16 and 17 were matched to cis-Δ9-palmitoleic acid and 3-hexadecenoic acid, respectively. The MS/MS spectra of these two fatty acids yielded molecular ions at [M − H]^−^ (*m*/*z* 253.2175) (*t*_R_ = 8.38) and [M − H]^−^ (*m*/*z* 253.2171) (*t*_R_ = 7.69).

These metabolites yielded fragment ions at *m*/*z* 217.1967 (C_16_H_25_) for cis-Δ9-palmitoleic acid and *m*/*z* 141.2954 (C_10_H_21_) for 3-hexadecenoic acid, respectively, indicating loss of [M − H − H_4_O_2_]^−^ and [M − H − C_6_H_8_O_2_]^−^ [22]. Peak 18 was assigned to stearidonic acid with a molecular ion at [M − H]^−^ (*m*/*z* 275.2015), and MS/MS fragments were observed at *m*/*z* 231.2124 (C_17_H_27_) due to loss of the CO group [22]. The metabolite at peak 19 at [M − H]^−^ (*m*/*z* 279.2331) was linoleic acid with deprotonated molecular ion gave fragment ions at *m*/*z* 111.3027 (C_8_H_15_) [M − H − C_10_H_16_O_2_]^−^ and *m*/*z* 59.0126 (C_2_H_3_O_2_) [M − H − C_16_H_28_]^−^, respectively [23].

The empirical molecular formula of the metabolites at peaks 20 and 21 were C_18_H_30_O_2,_ which matches the spectra peaks of the linolenic acid. However, to correctly determine the isomeric position of these acids whether they are α-linolenic acid (polyunsaturated *n*−3 (omega-3)) or γ-linolenic acid (polyunsaturated *n*−6 (omega-6)), analysis of standard should be done to confirm the results. These metabolites showed the same [M − H]^−^ (*m*/*z* 277.2173) based on common fragment masses *m*/*z* 69.9031 (C_5_H_9_) and 59.0124 (C_2_H_3_O_2_), due to the loss of [M − H − C_13_H_20_O_2_]^−^ and [M − H − C_16_H_26_]^−^ but with the difference peak intensity of these product ions present in the ESI tandem spectrum, suggesting the peak 20 is differ from peak 21 [21,24]. Peaks 22–23 were identified as arachidonic acid and eicosapentaenoic acid (EPA), this [M − H]^−^ *m*/*z* 303.2329 and [M − H]^−^ *m*/*z* 301.2174, respectively, based on their fragmentation spectra. Arachidonic acid had product ions at at *m*/*z* 287.0870 and *m*/*z* 259.2397 related to loss of a water molecule and a carbonyl group, while EPA with product ions *m*/*z* 273.5861 [M − H − C_2_H_4_]^−^ (28 amu) and *m*/*z* 257.2269 [M − H − COO]^−^ (44 amu) [21,23].

Peaks 24 and 25 were proposed as HEPEs isomers of EPA, and it could either be 5-HEPE and 15-HEPE [24]. The mass of metabolite at peak 24 are differing by 0.0004 Da from peak 25, with small differences in their exact masses, therefore we cannot be certain if these two isomers can be distinguish without good chromatography resolution mass spectra. It should be noted here that it was not possible to define the position of hydroxyl relative to the carboxylic group unless further work using commercial standard and exploring another advances method such as ultraviolet photodissociation (UVPD) in a hybrid MS^n^ to confirm these peaks identities. Both had molecular ions at [M − H]^−^ *m*/*z* 317.2124 and [M − H]^−^ *m*/*z* 317.2132, and both displayed fragment ions at *m*/*z* 255.2123 [M − H − CH_2_O_3_]^−^. Rettner et al. [24] presented that the fragment ion at *m/z* 115.0391 seemingly specific to the 5-HEPE MS/MS spectrum which related to the loss of [M − H − C_15_H_22_]^−^ and eluted later than 15-HEPE, therefore we used this information as guide to our identification. The property revealed that diatom cells of *C. calcitrans* exhibit a varied lipoxygenase (LOX) metabolism of EPA.

Docosahexaenoic acid (DHA) (*t*_R_ = 7.63) displayed [M − H]^−^ at *m*/*z* 327.2330 (peak 26) and its derivative, 4,5-epoxy-17*R*-HDHA, had [M + H]^+^ at *m*/*z* 399.2519 (peak 27) in positive ion mode [25]. The MS/MS spectrum of DHA displayed fragment ions at m/z 283.2432, corresponding to [M − H − COOH]^−^ (45 amu). 4,5-Epoxy-17*R*-HDHA was assigned based on the fragment ion at *m*/*z* 321.2201 (C_23_H_29_O), which is related to the loss of [M + H − C_2_H_6_O_3_]^+^. Peak 28 was identified as 6-bromo- Δ5-heptacosadienoic acid and was classified as a monosaturated bromo fatty acid [22]. The UHPLC-MS of the brominated fatty acid, 6-bromo-5-heptacosadienoic acid showed mass spectral peaks at m/z 485.3008 and 487.2993, which corresponding to the two isotopes of bromine. The product ion at *m/z* = 405.3321 is for the loss of the hydrogen bromide.

#### 2.1.3. Identification of Glycerolipids, Glycerolphospholipids and Sterol Lipid

MS/MS spectra interpretation in both ESI+ and ESI− permitted the identification of a total of 16 glycerollipids, glycerolphospholipids and 1 sterol lipid (Table 1). Despite the significant advances of the Orbitrap technology, it should be noted here that, differentiating structural isomers are remains challenging, especially for the complex lipids identified from biological mixtures, this approach does not precisely indicate the positions of the double bond and the stereochemistry. However, derivatization procedure and analyze by using GCMS is one of the promising methods to overcome this problem The high levels of phospholipids found in *C. calcitrans* are suggested to be related to the biosynthesis of PUFAs, namely, linoleic (18:2*n*-6), arachidonic (20:4*n*-6), *a*-linolenic (18:3*n*-3), eicosapentaenoic (20:5*n*-3) and docosahexaenoic (22:6*n*-3) fatty acids biosynthesis. The glycerophospholipids identified in positive ion mode spectra were C18: 3 Δ6, Δ9, Δ12/C19: 1 Δ9 phosphatidylinositol (PI), a phosphatidylinositol at peak 29; C16: 1 Δ9/C16:0 phosphatidylglycerol (PG) at peak 30 and C18: 3 Δ9, Δ12, Δ15/C13:0 phosphatidylglycerol (PG) at peak 31. Both of the latter are phosphatidylglycerol. C20: 1 Δ 11/C0:0 phosphatidic acid (PA), was detected at peak 32, and C18: 3 Δ6, Δ9, Δ12/C22:4 Δ7, Δ10, Δ13, Δ16 phosphatidic acid was detected at peak 35. Although the existing literature reports that PI is typically detected in negative ion mode [26], in this study PI, which displayed [M + H]^+^ at *m*/*z* 873.5486 can be clearly detected at peak 29 in the positive ion scan mode MS/MS experiment. The formation of a characteristic product ion at *m*/*z* 595.2532 (C_27_H_48_O_12_P), corresponded to the loss of the s*n*-1 or s*n*-2 substituent as a ketene yield ion, [M − H − C_19_H_34_O]^−^.

Metabolites at peak 30 and 31 were identified as C16: 1 Δ9/C16:0 phosphatidylglycerol (PG), C18: 3 Δ9, Δ12, Δ15/C13:0 phosphatidylglycerol (PG), respectively, based on their fragmentation spectra [27]. C16: 1 Δ9/C16:0 phosphatidylglycerol (PG) gave [M + H]^+^ ions at *m*/*z* 721.5011, had product ions at [M + H − C_4_H_10_]^+^ and [M + H − C_25_H_53_O_9_P]^+^ whereas C18: 3 Δ9, Δ12, Δ15/C13:0 phosphatidylglycerol (PG), [M + H]^+^ ion at *m*/*z* 703.4542 with product ions [M + H − CH_4_O]^+^ at *m*/*z* 671.4276 (C_36_H_64_O_9_P) and [M + H − C_3_H_8_O]^+^ at *m*/*z* 643.4357 (C_34_H_60_O_9_P). Peak 32 (*t*_R_ = 6.80) was tentatively assigned to C20:1 Δ 11/C0:0 phosphatidic acid (PA) with product ions [M + H − C_18_H_35_]^+^ and [M + H − C_5_H_11_O_6_P]^+^, at *m*/*z* 251.1775 (C_18_H_35_) and *m*/*z* 197.1311 [27,28]. Peak 35 gave protonated ion [M + H]^+^ at *m*/*z* 747.4981 (C_43_H_70_O_8_P), had product ions at *m*/*z* 731.1207(C_43_H_72_O_7_P) and 100.1120(C_5_H_8_O_2_) [29].

The metabolites at peaks 38, 43 and 44 exhibited the [M − H]^−^ ion at *m*/*z* 617.4211 (C_33_H_62_O_8_P), *m*/*z* 765.4713 (C_42_H_70_O_10_P) and *m*/*z* 790.5969 (C_43_H_85_NO_9_P) in the negative ion scan mode of the MS/MS experiment] and were identified as C18: 1 Δ9/C12: 0 phosphatidic acid, C16: 1 Δ9/C20: 5 Δ5, Δ8, Δ11, Δ14, Δ17 phosphatidylglycerol and O-C18:0/C19:0 phosphatidylserine [27,30,31,32].

Four diacylglycerols (DG) were identified; they are listed in Table 1. Peak 33 (*t*_R_ = 10.89 min) was C22: 3 Δ10, Δ13, Δ16/C22: 5 Δ7, Δ10, Δ13, Δ16, Δ19/C0: 0 diacylglycerol gave the [M + H]^+^ ion at *m*/*z* 721.5765 (C_47_H_76_O_5_, calculated 721.5765, error, −0.00 ppm) in the positive ion scan mode of the MS/MS mass spectrum [29]. The obtained ion produced of [M + H − C_25_H_41_O]^+^, [M + H − C_25_H_44_O]^+^ and [M + H − C_37_H_56_O_3_]^+^ fragments at *m/z* 316.5049, 313.1947 and 173.1183, in the MS/MS experiment. Peaks 39, 40 and 41 showed [M − H] ^−^ ions at *m*/*z* 655.5309 (C_42_H_71_O_5_, *t*_R_ = 13.62 min), *m*/*z* 663.5026 (C_43_H_67_O_5_, *t*_R_ = 6.66 min) and *m*/*z* 719.5636 (C_47_H_75_O_5_, *t*_R_ = 18.04 min), respectively [28,29]. The obtained ion produced of *m/z* 316.5049 [M + H − C_25_H_41_O]^+^, *m/z* 313.1947 [M + H − C_25_H_44_O]^+^ and *m/z* 173.1183 [M + H − C_37_H_56_O_3_]^+^ represent the possible structure for peak 39, C17: 0/C22: 5 Δ7, Δ10, Δ13, Δ16, Δ19/C0: 0 diacylglycerol. Peak 40 was C20: 3 Δ8, Δ11, Δ14/C20: 5 Δ5, Δ8, Δ11, Δ14, Δ17/C0: 0 diacylglycerol, had product ions at *m*/*z* 375.2085 (C_23_H_35_O_4_) [M − H − C_20_H_32_O]^−^. Metabolite at peak 41 has [M − H − C_4_H_8_]^−^ and [M − H − C_19_H_35_O]^−^ ions at *m*/*z* 664.5043 (C_43_H_68_O_5_) and 440.8061 (C_19_H_35_O), respectively, which confirmed it as C22: 2 Δ13, Δ16/C22: 6 Δ4, Δ7, Δ10, Δ13, Δ16, Δ19/C0: 0 diacylglycerol (DG) [27,29,30].The observed peaks for DG might be explained with the limitation of phosphates, which similarly occurred in plants. Due to phosphorus (P) limitation, the glycerolphospholipids, PC and PI are catalyzed by phospholipases to generate phosphocholine, phosphoinositol, and diacyglycerol (DG), respectively [33]. A triacylglycerol (TG) found at peak 34 was identified as C18: 3 Δ9, Δ12, Δ15/C19: 1 Δ9/C20: 5 Δ5, Δ8, Δ11, Δ14, Δ17 triacylglycerol (TG). The ESI-MS/MS spectrum of peak 34 showed [M − H − C_31_H_56_O]^−^ ions at *m*/*z* 469.3639 [6]. Similarly, the detection of TG could result from degradation of phospholipid under P limitation after the enzymes involved in TG biosynthesis are unregulated (i.e., Kennedy pathway) [33].

Other types of glycerolipids were found in the ESI-MS/MS spectrum of *C. calcitrans* at peaks 42 and 45 were C16:0/C18: 2 Δ9, Δ12 diacylglyceryl-*N*,*N*,*N*-trimethyl homoserine (DGTS) and C16:0/C16:0 sulfoquinovosyl diacylglyceride (SQDG), tentatively identified based on previously reported literature [31,33]. Sterols are essential dietary nutrients for most microalgae. A sterol lipid, 5-androstenetriol at peak 37 (*t*_R_ = 3.06 min) was also detected in *C. calcitrans* based on [M + H]^+^ ion at 307.2272 (C_19_H_31_O_3_). The obtained ion produced the [M + H − C_3_H_8_O_3_]^+^ fragment of 5-androstenetriol at *m*/*z* 215.1790 (C_16_H_23_) in the MS/MS spectrum. Although there is still insufficient information about this mid-chain hydroxyl group at position C-19 type of sterol in *C. calcitrans*, but they are widespread and abundant in marine sediments [29].

#### 2.1.4. Identification of Other Possible Compounds

Other possible metabolites such as crocetin dialdehyde (C20 isoprenoid (diterpene) derivatives, peak 46), *N*-butyl-*N*(2)-[(*R*)-2-hydroxy-2-phenylacetyl]-l-serinamide (*N*-acyl-alpha amino acids and derivatives, peak 47), formyl 2,4,6-decatrienoate (peak 48), while peaks 49 and 50 were tentatively identified and characterized as dipentyl phthalate and diisooctyl phthalate (benzoid acid esters derived from the oxidation of fatty acids), 9, 11 pentadecadienal (peak 51) were tentatively identified based on previously reported data [6,30,34,35,36,37]. The identity of metabolites at peaks 52, 53, and 54 were identified as fatty amides resulting from McLafferty rearrangement of γ-cleavage, and compared with the information in the online database [6].

### 2.2. Chemometric Analysis of MS Data

#### 2.2.1. Discrimination through Unsupervised Principal Component Analysis (PCA)

**Figure 3:**
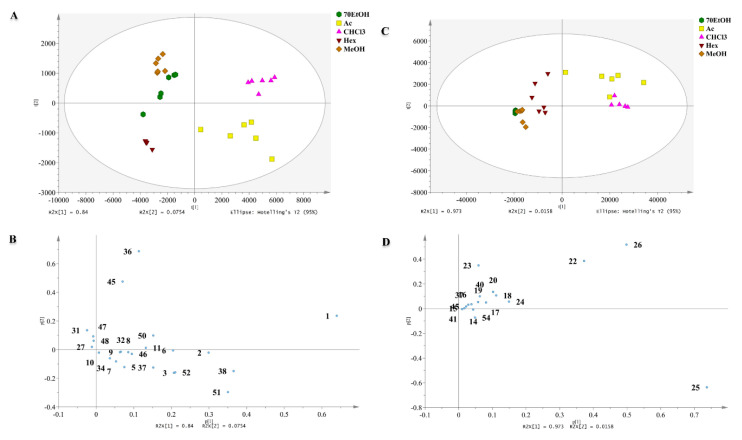
(**A**) Score plot (**B**) loading plots of two-dimensional principal component analysis (2D-PCA) in the microalga diatom *C. calcitrans* in positive ionisation. (**C**) Score plot (**D**) loading plots of two-dimensional principal component analysis (2D-PCA) in the microalga diatom *C. calcitrans* in negative ionisation (Ac) Acetone, (CHCl_3_) Chloroform, (Hex) Hexane, (MeOH) Methanol and (70EtOH) 70% Ethanol.

Metabolomics provides an alternative strategy to efficiently evaluate high volume information found in UHPLC-ESI-Orbitrap MS analysis. Therefore, for interpreting the datasets of metabolome, a mathematical and statistical method such as chemometrics is required. Scatter plot of PCA suggests the relative variability and similarity among samples whereas the PCA loading plot reveals the area of the spectra that lead to the clustering plot. In this section, the web-based platform XCMS Online was employed, and the extracted mass signals obtained in both positive and negative ionization modes were then subjected into chemometrics analysis. The results for both scan modes showed a clear variation through two principal components (PC1 and PC2). The first two PC accounted 91.54% of total variance with chloroform and acetone extracts clustered in the lower and upper chamber of right quadrant, whereas the rest of the extracts were observed on the left side. The ethanol and methanol extracts were clustered together in the positive PC2 upper chamber while hexane extract clustered in the negative side of PC2 (Figure 3A). The PCA score plot modeled from negative ionization mode accumulated variance of 98.88% from the original data, and demonstrated that chloroform and acetone extracts were correlated and separated from the rest of extracts by PC1 and PC2 (Figure 3C). In PCA score plots, chloroform and acetone extracts were observed in the right side, but the position was switched either in positive or negative quadrant of PC1.

The segregation profiles of each extract observed in scatter plot directly contribute to the distribution of variables positioned in the same dimension in PCA loading plot. The distance of variables from the plot origin is useful for interpretation. The further away the points of variable from the (0, 0) coordinates either negatively or positively, the stronger their influence on the model. As revealed in Figure 3B run in positive ion mode, the notable predominant metabolites fucoxanthin (1), C18: 1 Δ9/C12: 0 phosphatidic acid (PA) (38), C20: 5 Δ5, Δ8, Δ11, Δ14, Δ17/C15: 0 phosphatidylcholine (PC) (36) and 9E,11Z pentadecadienal (51) contributed positively to PC1, while MS peak for PG (18:3(9,12,15)/13:0) contributed negatively to PC1. On the other hand, the PCA loading plot for negative ion mode revealed the influential metabolites to segregation pattern included arachidonic acid (22), eicosapentaenoic acid (23), 15-HEPE (25), and docosahexanoic acid (26) (Figure 3D).

#### 2.2.2. Correlation of the Metabolites with Antioxidant and Anti-Inflammatory Activities Using Partial Least Square (PLS) Regression Analysis

**Figure 4:**
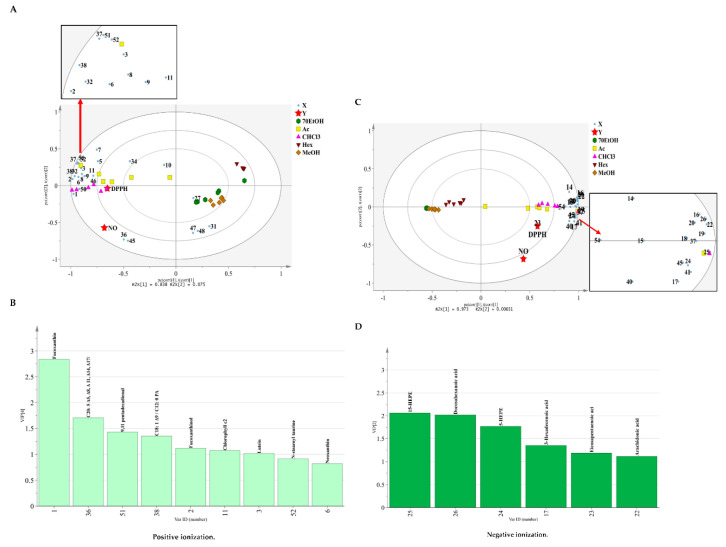
The PLS analysis for (**A**) correlation between identified metabolites in positive ionization (mass signals data) with biological activities (**B**) The variable importance in the projection (VIP) values (≥1, positive mode) (**C**) The PLS analysis showing correlation between identified metabolites in negative ionization based on the mass signals obtained from UPLC-MS analysis and the biological activities (**D**) The variable importance in the projection (VIP) values (≥1, negative mode). NO: NO inhibitory; DPPH: DPPH scavenging activities. (Ac) Acetone, (CHCl_3_) Chloroform, (Hex) Hexane, (MeOH) Methanol and (70EtOH) 70% Ethanol.

Subsequently, PLS was employed by merging the information of the peaks of MS spectra as X variables and the biological activities as Y variables. In addition, variable importance in the projection (VIP) was used to mark the X variables that had influenced in the PLS model as shown in Figure 4B,D. The PLS biplot obtained from positive ion mode exhibited the values of total sum-of-variation in Y explained by the model, *R*^2^Y = 0.795 with good predictive ability with *Q*^2^ = 0.715 whereas the PLS model acquired from negative ion mode dataset was statically adequate with values *R*^2^Y = 0.788 and goodness of prediction, *Q*^2^ = 0.691. The PLS biplot models were formed in a similar way as those exhibited in PCA (Figure 4A,C). The following discussion will be examined in the sequence of high to low significant of the groups and metabolites in the tested bioactivities. The notable metabolites found in positive ion mode included 9,11 pentadecadienal (51), C18: 1 Δ9/C12: 0 phosphatidic acid (PA) (38) and fucoxanthinol (2), were ranked in the top five highest among the X variables analyzed in VIP values list, were found in chloroform and acetone extracts, were found to have positive correlation with DPPH free scavenging activity. Fucoxanthin (1) and C20: 5 Δ5, Δ8, Δ11, Δ14, Δ17/C15: 0 phosphatidylcholine (PC) (36) (compounds with VIP value ≥ 1.0) which were also well correlated with chloroform and acetone extracts, were located in the negative side of PC2 and therefore were negatively correlated with the observed *Y* variables. These metabolites are valuable to evaluate as they might provide indirect effect on reducing the tested bioactivities. 15-HEPE (25), docosahexaenoic acid (26), 5-HEPE (24), eicosapentaenoic acid (23), and 3-hexadecenoic acid (17) (VIP value ≥ 1.0) were obtained in negative ion mode of MS spectra. They were occupied in chloroform and acetones extracts either in lower or upper chamber and were positioned close to the Y variables. The metabolites had positive correlations with bioactivities were 15-HEPE (25), 5-HEPE (24), and 3-hexadecenoic acid (17). Note that, from the PLS biplot, no tentatively identified metabolites were observed in the hexane extract, which might indicate that this extract was inactive towards the bioactivities. Both models were shown to have a satisfactory level of validity as evidenced by the results of permutation test and regression analysis (Supplementary Figures S5 and S6).

**Figure 5:**
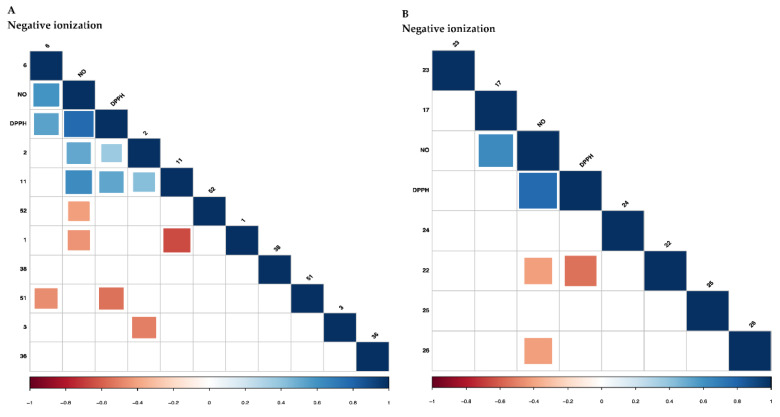
Correlogram visualizing correlation between metabolites analyzed using UHPLC-ESI-Orbitrap MS analysis and biological activities. Correlation with *p*-value > 0.05 are considered insignificant and are represented by the blank white space. Color and size of the squares are proportional to the correlation coefficients. Positive correlations are shown in blue (different shades; dark blue with the strongest correlation) whereas negative correlations in red (ranging from light red to medium red; dark red with the weakest correlation). Assignment of metabolites: (**A**) Positive ionization UHPLC-MS: 6, Neoxanthin; 2, Fucoxanthinol; 11, Chlorophyll c2; 52, *N*-stearoyl taurine; 1, Fucoxanthin; 38, C18: 1 Δ9/C12: 0 phosphatidic acid (PA); 51, 9,11 pentadecadienal; 3, Lutein; 36, C20: 5 Δ5, Δ8, Δ11, Δ14, Δ17/C15: 0 phosphatidylcholine (PC); (**B**) Negative ionization UHPLC-MS: 23, Eicosapentaenoic acid; 17, 3-Hexadecenoic acid; 24, 5-HEPE; 22, Arachidonic acid; 25, 15-HEPE; 26, Docosahexaenoic acid.

The correlation was further evaluated by computing the Pearson correlation and the correlogram plot were then produced (Figure 5) to confirm the strength of correlation with several variables. Only metabolites with values ≥ 1.0 were highlighted for both scan modes, which in total 15 metabolites were selected. The obtained correlogram showed strong and significant correlations for fucoxanthinol (2) and chlorophyll c2 (11) with the two biological activities (Figure 5A). The free radical scavenging capacities of these metabolites have been revealed in previous studies [38,39,40]. Further, 3-Hexadecenoic acid (17) was found to be significantly correlated with NO inhibitory activity but not with DPPH. Meanwhile, arachidonic acid (22) was weakly linked (light red) with NO inhibitory and DPPH activities (Figure 5B). Docosahexaenoic acid (26) was also found to weakly link with NO inhibitory activity (Figure 5B).

### 2.3. Quantification of the Metabolites in Microalgal Diatom C. calcitrans Extracts through UHPLC-ESI-Orbitrap MS

#### 2.3.1. Relative Quantification of Metabolites in *C. calcitrans*

**Figure 6:**
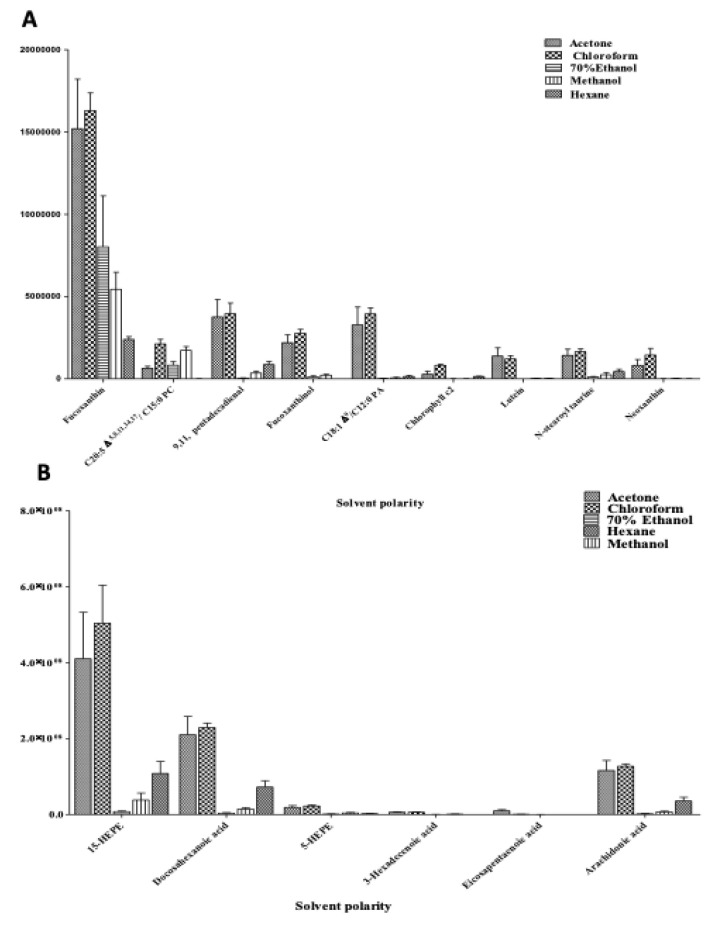
The Relative quantification of the identified compounds (**A**) Positive ionization UHPLC-MS: 6, Neoxanthin; 2, Fucoxanthinol; 11, Chlorophyll c2; 52, *N*-stearoyl taurine; 1, Fucoxanthin; 38, C18: 1 Δ9/C12: 0 phosphatidic acid; 51, 9,11 pentadecadienal; 3, Lutein; 36, C20: 5 Δ5, Δ8, Δ 11, Δ14, Δ17/C15: 0 phosphatidylcholine; (**B**) Negative ionization UHPLC-MS: 23, Eicosapentaenoic acid; 17, 3-Hexadecenoic acid; 24, 5-HEPE; 22, Arachidonic acid; 25, 15-HEPE; 26, Docosahexaenoic acid.Data presented are based on the mean of six replicates each of the solvent systems (acetone, chloroform, hexane, methanol, 70% ethanol) ± standard deviation (SD).

A total of 15 metabolites (positive ion mode = 9 and negative ion mode = 6) with VIP scores ≥ 1.0 were relatively quantified using SPSS 16.0 and are displayed in bar chart to further support the obtained results. The intensity of the metabolites was calculated based on the mean peak area generated from MS spectra. The discussion focuses on the chloroform and acetone extracts, as most metabolites that contribute to the measured bioactivities are highly concentrated in both extracts. The extracts contained high level of fucoxanthin, C20: 5 Δ5, Δ8, Δ11, Δ14, Δ17/C15: 0 phosphatidylcholine (PC), 9,11 pentadecadienal, C18: 1 Δ9/C12: 0 phosphatidic acid (PA), fucoxanthinol, chlorophyll c2, *N*-stearoyl taurine and neoxanthin, 15-HEPE, docosahexanoic acid, 5-HEPE and arachidonic acid as shown in Figure 6A,B. Fucoxanthinol and chlorophyll c2 were found to be significantly abundant (0.010 ≥ *P* > 0.001) in extracts of chloroform and acetone. This was supported by previous study that also identified the fucoxanthinol from medium polar extract of *C. calcitrans* [39,40]. On the contrary, chlorophyll c2 has been identified from the buffer extract [5]. Additionally, chloroform and acetone extracts contained a comparable amount of (E)-3-hexadecenoic acid (not significant, *p* ≥ 0.005) and eicosapentaenoic acid (highly significant, *p* ≤ 0.001) among others (Supplementary Table S1). It is notable that the extract prepared from medium polar solvent could retain a large number of hydrophobic molecules.

By relating the relative quantification data with PLS biplot and correlogram, fucoxanthinol, chlorophyll c2 and 3-hexadecenoic acid in chloroform and acetone extracts were located on the positive sides of PC2 in the PLS biplot with strong and significant correlations. Moreover, it is noted that the content of these metabolites was slightly higher in chloroform extract than in acetone extract. Overall, these metabolites might be the main contributors to the measured bioactivities.

#### 2.3.2. Validation of the Proposed Method

Validation of the analytical method was performed to exclude interference or endogenous substance in a tested sample, ensuring the accurate identification and quantification of the targeted metabolites. In this part, ESI positive ion mode was used as the four-targeted metabolites were tentatively identified in this scan mode. Parameters such as linearity, limit of detection (LODs) and limit of quantification (LOQs) were evaluated according to FDA guideline on bioanalytical method validation [41].

##### Linearity

The linearity was measured by injecting different level of concentrations for each standard over the range 1.25–125 μg/mL. Linearity was determined based on the least-square linear regression. In the calibration curve equations for fucoxanthin (*y* = 0.0000095*x* − 2.0495757; *R*^2^ = 0.9967099), astaxanthin (*y* = 0.000645253*x* + 0.422860969; *R*^2^ = 0.9998498), zeaxanthin (*y* = 0.0001478*x* + 2.9163077; *R*^2^ = 0.9966875), and lutein (*y* = 0.0142099*x* − 1.0372580; *R*^2^ = 0.9990715), where *y* represents the peak area corresponding to the concentrations of analyte, and *x* represents the known concentration of the standard compound. Excellent linearity can be seen with determination coefficients higher than 0.98 (Table 2), using peak area as the analytical response.

##### LOD and LOQ

LODs and LOQs were defined as the lowest concentration levels injected which yielded signal-to-noise (S/N) ratios of 3 and 10, respectively, at which the concentration is reliable for differentiating the signal produced by the analyte peak from the background noise. The LODs ranged from 4.68335 × 10^−8^ mg/mL to 0.000881644 mg/mL, and the LOQs ranged from 1.56112 × 10^−7^ to 0.002938813 mg/mL (Table 2). These values were adequate for confirmation of the quantity of the targeted metabolites in real matrix samples.

##### Application of Method: Quantification of the Targeted Metabolites in Active Extracts of *C. calcitrans*

The optimized UHPLC–MS/MS method was then applied to chloroform and acetone extracts of the microalgal diatom *C. calcitrans* and the quantitative results are shown in Table 3. The present method detected relatively high concentration values for fucoxanthin in the extract in comparison with astaxanthin, zeaxanthin and lutein. The observed results for the concentration of fucoxanthin are consistent with the results of a previous study in which the concentrations of these compounds were measured by the HPLC technique [42]. The compound present at the second highest concentration was astaxanthin, followed by lutein and zeaxanthin.

## 3. Materials and Methods

### 3.1. Chemicals and Samples

All aqueous and organic solvents used were LC/MS grade. Water, acetonitrile and methanol were obtained from Thermo Fisher Scientific (Bremen, Germany). Ammonium formate and formic acid were also supplied by Thermo Fisher Scientific (Bremen, Germany). The reference standards of carotenoids: (1) fucoxanthin, (2) astaxanthin, (3) zeaxanthin, and (4) lutein were purchased from ChromaDex, Inc. (Irvine, CA, USA). All chemicals and solvents were of analytical grade or higher purity. The microalga *Chaetoceros calcitrans* was supplied by Laboratory of Marine Biotechnology, Institute Biosciences, Universiti Putra Malaysia, where they obtained the algal biomass from Pantai Morib, Selangor. Stocks of the microalga were kept in 500 mL borosilicate glass fitted with cotton wool bungs. All stock-cultures were regularly cultivated every three to four weeks.

The microalga biomass production was accomplished in batch culture systems consisting of 5 L modified plastic bottle equipped with inlet and outlet tubes for aeration. The microalga was grown with aeration with mixture of air (at rate 0.2 L/min) and CO_2_ (containing 1–2%) in presence of light (double fluorescence lamp unit, 300 μmol photon m^−2^s^−1^, 18 hrs of light) at room temperature (25 °C). During the culture for 7 days, the pH of the medium was maintained below 8.0 by the bubbling of air containing CO_2_. These are important factors for better growth condition. Besides, the microalga biomass production was cultivated in the autoclaved pure seawater with a salinity of 30 parts per thousand (ppt) on a standard f/2 media that comprised of: filtered natural seawater, monosodium phosphate (NAH_2_PO_4_.2H_2_O), and sodium nitrate, sodium metasilicate nonahydrate (Na2SiO_3_.9H_2_O), trace metal solution (ferric chloride hexahydrate (FeCl_3_.6H_2_O), EDTA disodium dehydrate, (Na_2_EDTA. 2H_2_O), sodium molybdate dehydrate (Na_2_MoO_4_.2H_2_O), zinc sulfate heptahydrate (ZnSO_4_.7H_2_O), copper(II) sulfate pentahydrate (CuSO_4_.5H_2_O), cobaltous chloride hexahydrate (CoCl_2_.6H_2_O), manganese(II) chloride tetrahydrate (MnCl_2_.4H_2_O) as well as vitamin solution (distilled water, thiamine HCl (Vitamin B1), biotin (Vitamin B7), cyanocobalamin (Vitamin B12). Solution B (zinc chloride, cobaltous chloride, ammonium molybdate, cupric sulphate, concentrated HCl, filtered natural; water), Solution C (Vitamin B1, Vitamin B12, fresh water), and Solution D (sodium metasilicate nonahydrate, fresh water) for the growth of microalga. The whole growth medium was adjusted to pH 8.

After the cultures were allowed to grow till 5–8 days (end of exponential phase/start of the stationary phase) and considered achieved highest densities (0.5 DW (g per L^−1^)), this alga biomass was harvested by centrifugation using high-speed Sorvall Evolution RC centrifuge (Thermo Electron Corporation, NC, USA) at speed 12,000 rpm at 4 °C, for 5 min, necessary for sedimentation. After centrifugation, pellets were washed by resuspension in distilled water and repeated the centrifugation to free of from remaining salts. Then, the harvested microalga was freeze-dried by using ScanVac CoolSafe Freeze DryerTM (Labogene, Lynge, Denmark) kept at −80 °C. The extract was kept in dim light at −20 °C until use. The grounded microalga powder was then subjected to five different solvent extractions including 100% chloroform, 100% acetone, 100% methanol, 70% ethanol, and 100% hexane and ultrasound assisted extraction was applied. In each sample, the microalga sample was kept constant, i.e., 100 mg of microalga sample in 50 mL of solvent. Sonication was carried out of 30 min using an ultrasonic water bath (SK8210HP, Shanghai KUDOS Ultrasonic Instrument Co. Ltd., China) at room temperature. The extraction procedure was repeated three times for each sample. Crude extracts were then filtered, vacuum-evaporated, freeze-dried, and kept in a chiller (−20 °C) until further analysis.

### 3.2. In Vitro Preliminary Assays

#### 3.2.1. 2,2-Diphenyl-1-picrylhydrazyl (DPPH) Radical Scavenging Assay

The antioxidant potential of microalga diatom *C. calcitrans* was determined based on the DPPH free radical scavenging assay according to the method reported in our previous study [3]. Each sample was prepared in dimethyl sulfoxide (DMSO) at a stock concentration of 1000 ppm. Quercetin was used as standard. The results were expressed as a percentage of DPPH inhibition.

#### 3.2.2. Nitric Oxide (NO) Inhibitory Assay

The nitric oxide inhibitory by *C. calcitrans* extracts was determined using the Griess assay as described in detail in our previous study [3]. The RAW 264.7 cell lines were incubated at 37 °C in Dulbecco’s Modified Eagle’s medium (DMEM) media supplemented with 10% (v/v) inactivated fetal bovine serum and 100 U/mL penicillin/streptomycin under a water-saturated atmosphere of 95% air and 5% CO_2_. The cells were seeded in 96-well culture plates (5 × 10^4^ cells/well) and allowed to adhere for 17 h at 37 °C. The cells were incubated with 10 μg/mL of LPS and 200 units/mL of recombinant murine IFN-_γ_ in the absence or presence of the test compounds. Cucurmin was used as a positive control. To quantify the NO synthesis, the nitrite concentration was measured by the Griess reaction using the supernatant of the RAW 264.7 cell. The remaining cells in the incubated wells were measured for cell viability using 3-(4,5-dimethylthiazol-2-yl)-2,5diphenyltetrazolium bromide (MTT) reagent.

### 3.3. Targeted Metabolite Profiling Using UHPLC-ESI-Orbitrap MS

#### 3.3.1. Sample Preparation of UHPLC-ESI-Orbitrap MS Approach

The sample extracts were dissolved in LCMS grade methanol respectively, at concentrations 2 mg in 1 mL. Then, the dissolved extract was vortexed for 3–4 min and sonicated for 5 min. All the dissolved microalga extracts were filtered through a 0.22 μm Nylon membrane filter (Millipore, Billerica, MA, USA) before UHPLC-MS analysis.

#### 3.3.2. UHPLC-ESI-Orbitrap MS Analysis

Chromatographic separation was performed on ultra-high performance liquid chromatography (UHPLC) system consisted of an Ultimate 3000 LC system (Thermo Scientific™ Dionex™, Sunnyvale, CA, USA), to a surveyor UHPLC binary pump, a photodiode array detector (PDA) detector, scanning from 200 to 600 nm and auto-sampler (Exactive^TM^, Thermo Fisher Scientific, Bremen, and Germany). High-resolution MS analysis was carried out using Thermo Fisher Scientific™ Q Exactive™ Focus Quadrupole-Orbitrap mass spectrometer (Thermo Fisher Scientific, San Jose, CA, USA) operated at 70,000 resolutions (FWHM), which was equipped with a heated electrospray ionisation (HESI II) source. The UHPLC-ESI-Orbitrap MS system was controlled by Exactive Tune 1.1 and Xcalibur 2.0 software (Thermo Fisher Scientific, San José, CA, USA).

This following procedure was performed in accordance with the procedure described by Fu, et al. [43] and Bijttebier, et al. [18], with modifications. An analytical column used were ACQUITY UPLC HSS T3 1.8 μm (2.1 × 150 mm) (Waters, Manchester, UK). The flow rate was 0.25 mL/min and the column temperature were maintained at 30 °C. The mobile phase consisted of eluent A, a mixture acetonitrile (ACN)/methanol (MEOH)/2-propanol (70:20:10, *v*/*v*/*v*) containing 10 mM ammonium formate with 1% formic acid, and eluent B, 10 mM ammonium formate with 1% formic acid in water. The following gradient was used: 0 min, 70% A; 1.17 min, 70% A; 7 min, 90% A; 9.73 min, 100% A; 30 min, 100% A, 31 min, 70%A; 35 min; 70%A until the end of the run at 30 min [44,45]. The sample injection volume was set at 2 μL. All the tentatively identified analytes eluting over 0–30 min while the last 5 min were used for column cleaning and re-equilibration.

The ESI source was operated in the positive full scan mode and the parameters were as follows: sheath gas flow rate of 50 arbitary (arb) unit, auxillary gas flow rate of 10 arbs, electrospray voltage of 4.0 kV, capillary temperature of 350 °C, heater temperature of 300 °C, S-lens RF level of 55, the collision energy of 10%. Mass spectrometric analysis subsequently was performed for data-dependent acquisition (DDA) with priority to mass-to-charge ratios (*m/z*) of parent compounds and their expected metabolites (separate inclusion lists for fucoxanthin, astaxanthin, lutein, and zeaxanthin). Discovery mode was chosen to ensure the recording of MS^2^ spectra of precursor ions not in the inclusion list. The settings for full-scan data acquisition were as follows: polarity, positive; resolution, 70,000; scan range, *m/z* 100–1500; automatic gain control (AGC) target, 1e6; auto maximum injection time (IT); microscans, 1; spectrum data type, profile. The settings for the DDA mode were as follows: dd-MS^2^, discovery; resolution, 17,500; isolation window, 3.0 *m/z*; AGC target, 5e4; maximum injection time, 250 ms; high-collision dissociation cell with stepped normalized collision energy, 10; loop count, 1; minimum AGC target, 8e3 (corresponds to auto signal intensity threshold); exclude isotopes, on; and spectrum data type, profile. Mass calibration was conducted prior to analysis according to the manufacturer’s recommendations using external mass calibration. The same experiment was also performed in the negative ion mode. Data were acquired and analyzed using Xcalibur 2.0.7 software (Thermo Fisher Scientific). The option of “all ion fragmentation” using the high-energy collision dissociation (HCD) cell was applied to investigate the confirmation potential of generated fragments and was turned on during the actual analysis [46].

Metabolites were tentatively identified based on retention time, UV-vis spectral characteristic features and with the exact mass measurement. The identification of metabolites was further confirmed by comparing the retention time and MS/MS spectra with commercially available standards or matching accurate mass and fragment information with Metabolomics workbench (http://www.metabolomicsworkbench.org/), MetFrag (https://msbi.ipb-halle.de/MetFrag/), METLIN (http://metlin.scripps.edu), GNPS (https://gnps.ucsd.edu/), ReSpect (http://spectra.psc.riken.jp/) and HMDB (http://www.hmdb.ca/) databases. The exact *m/z* and retention time of each metabolite were used for targeted metabolomic analyses using the single ion monitoring (SIM) by setting for a narrower mass range as provided in Xcalibur software package. Furthermore, the identification of the metabolites was further supported by the mass error calculations (reported as ppm) whereby the lowest values suggested higher probability for an accurate metabolite identification and vice-versa. Quality control (QC) were performed once every ten of LCMS runs to monitor variation, quality of the MS data being generated and reproducibility of the analytical instrument. This method is extremely worthwhile as QC was used to check for intensity of peak signals and drift of the retention time.

#### 3.3.3. UHPLC-ESI-Orbitrap MS Data Preprocessing

The raw data were first transformed into centroid mode data of mzXML format using MSConvert tool from Proteowizard program (http://proteowizard.sourceforge.net/). Then data pretreatment which include non-linear retention time correction, peak filtration, calibration, extraction, peak picking and matching was performed by using open source XCMS online software (https://xcmsonline.scripps.edu/). Data were processed by using the following parameters as recommended for UHPLC (Q-Exactive) [47,48].

The obtained peak lists of the datasets were normalized to the total integrated area per sample in Microsoft Excel. The region from retention time 0 to 0.98 min was excluded from the data results because not significant for further metabolome analyses. Data result sets containing all the m/z value of UHPLC-ESI-Orbitrap MS datasets, retention time and ion peak area of each sample, which corresponds to the intensity of certain metabolite, were exported to the multivariate statistical software SIMCA (version 14.0, Umetrics, Umea, Sweden) for the subsequent unsupervised and supervised analysis, including partial component analysis (PCA) and partial least square analysis (PLS).

### 3.4. Targeted Metabolites Quantification Using UHPLC-ESI-MS/MS Orbitrap

#### 3.4.1. Relative Quantification of the Identified Metabolites Using UHPLC-ESI-MS/MS Orbitrap Approach

Relative quantification of the identified metabolites was measured using their representative mean peak area of the MS signals, after preprocessing method. The chromatographic peaks in MS data with distinct m/z values, but at similar retention times was assigned to define each metabolite of interest. The changes in metabolite levels of different solvent extractions were quantitatively evaluated and displayed in bar chart using SPSS 16.0 software (IBM, Chicago, IL, USA) and GraphPad Prism Version 5.01 (GraphPad Prism Software Inc., San Diego, CA, USA). Tukey’s significant difference multiple-comparison test was performed to evaluate the significant differences between the extracts.

#### 3.4.2. Targeted Metabolites Quantification Using UHPLC-ESI-MS/MS Orbitrap Analysis

A total of four standards including fucoxanthin, astaxanthin, zeaxanthin and lutein were used. Each standard with its precursor and fragment ions predefined by running full scan mass acquisition in positive ion mode from *m*/*z* 100 to 1500. These data were used to search the appropriate retention time windows (RTWs) for confirming the presence and the mass spectral identity of the targeted carotenoids in the extract. The setting for ESI interface parameters were similar as performed in Section 2.3.2. The injection volume for each standard was set to 2 μL and they were prepared at 5–10 serial dilutions in a screw-capped amber glass vials to plot the calibration curve (ranging from 1.25–125 μg/mL). For calculating the concentration of each targeted carotenoid compound in real samples (microalga extracts), the area under the peak was used and expressed as micrograms per milligram of microalga extract.

### 3.5. Statistical Analysis

Results are presented as the mean of six replicates ± SD. Statistical significance between groups was determined by one-way ANOVA. The significant level was established at *p*-value set at *<* 0.05.

## 4. Conclusions

Metabolomics approach using high resolution UHPLC-ESI-Orbitrap MS has proven to be an effective tool in evaluating variation among different microalga extracts. A total of 54 metabolites were unambiguously or tentatively identified. The UHPLC-ESI-Orbitrap MS allowed the additional information for the structural characterization of targeted metabolites. However, a comparison of the data with the available databases remains insufficient for identification due to almost identical positional isomers. The only confident way for complex structural analysis for identification metabolites and proposed structures are by using methods such as NMR spectroscopy, methyl esterification (FAME) and GCMS analysis. The present study proposes that extracts prepared from chloroform extract followed by acetone extract had stronger DPPH free radical scavenging capacities and inhibition effect of NO production than extracts prepared from the other solvents. Chemometric analysis and quantification by UHPLC-ESI-MS/MS approaches suggest that fucoxanthinol, chlorophyll c2, and 3-hexadecenoic acid in chloroform and acetone extracts were the main contributors to the antioxidant and anti-inflammatory properties of microalga. Although the targeted metabolites included fucoxanthin, lutein, zeaxanthin and astaxanthin were not the main bioactivity contributors, their synergistic effect towards the bioactivity should not be disregarded. This study facilitates the quality control and pharmaceutical authentication of *C. calcitrans* for safe and efficacious use in the food supplement industry and in producing feed for animals in marine aquaculture food products, and therapeutic functions. It could also provide a basis for further in vivo studies of *C. calcitrans*.

## Figures and Tables

**Table 1:** Metabolites identified from *C. calcitrans* by UHPLC-ESI-Orbitrap MS.

Peak no.	*t*_R_ (min)	Tentative Identification	Ion Mode	Molecular Formula	UV	[M + H]^+^/[M − H]^−^ *m*/*z*			Fragment Ions of [M + H]^+^/[M − H]^−^	Ref.
						Measured Mass (Da)	Theoretical Mass (Da)	Mass Error (ppm)		
1	8.19	Fucoxanthin	ESI+	C_42_H_58_O_6_	412,442	659.4294	659.4306	−1.82	641.4180, 581.3969, 489.2938	[16,17]
2	5.9	Fucoxanthinol	ESI+	C_40_H_58_O_5_	224,426	617.4172	617.4200	4.53	599.4075, 581.3972,	[17]
3	10.54	Lutein	ESI+	C_40_H_56_O_2_	226,408	569.4210	569.4280	−12.29	523.5612, 495.3164	[18,19]
4	10.52	Zeaxanthin	ESI+	C_40_H_56_O_2_	226, 408	569.4204	569.4280	−13.34	523.5612.	[18,19]
5	9.56	(3′R,3′S)-Astaxanthin	ESI+	C_40_H_52_O_4_	474	597.3903	597.3938	−5.85	147.1163, 173.1319, 201.1262	[19]
6	6.73	Neoxanthin	ESI+	C_40_H_56_O_4_	400, 420	601.4238	601.4251	−2.16	583.4016	[6,18]
7	9.75	Diadinoxanthin	ESI+	C_42_H_54_O_3_	404	583.4135	583.4145	1.71	565.4039, 547.3956	[6,18]
8	9.29	(3,4,3′)-4-Hydroxy-alloxanthin	ESI+	C_40_H_52_O_3_	408	581.3970	581.3989	−0.69	563.3879	[6]
9	8.76	Canthaxanthin	ESI+	C_40_H_52_O_2_	408	565.4026	565.4040	−2.48	547.3898	[18]
10	9.23	14′-apo-beta-carotenal	ESI+	C_22_H_31_O	414	311.2369	311.2369	0	293.2261	[18]
11	6.88	Chlorophyll c2	ESI+	C_35_H_28_MgN_4_O_5_	424	609.1981	609.1983	−0.33	591.1877, 549.1774	[20]
12	25.49	Pheophytin a	ESI+	C_55_H_74_MgN_4_	506,536	871.5694	871.5732	4.36	593.2754	[20]
13	19.26	Pheophytin b	ESI+	C_55_H_74_MgN_6_	536	885.5518	885.5525	0.79	593. 533	[20]
14	7.31	Myristic acid	ESI−	C_14_ H_28_ O_2_	N.D	227.2104	227.2107	−1.32	191.4482	[21]
15	8.58	Palmitic acid	ESI−	C_16_ H_32_ O_2_	N.D	255.2330	255.2330	0	61.98716	[22,23]
16	8.38	cis-Δ9-Palmitoleic acid	ESI−	C_16_ H_30_ O_2_	N.D	253.2175	253.2173	0.79	217.1967	[22]
17	4.96	3-Hexadecenoic acid	ESI−	C_16_ H_30_ O_2_	N.D	253.2171	253.2173	1.58	141.2954	[22]
18	6.27	Stearidonic acid	ESI−	C_18_ H_28_ O_2_	N.D	275.2015	275.2017	0	231.2124, 217.8763	[21]
19	8.39	Linoleic acid	ESI−	C_18_ H_32_ O_2_	N.D	279.2331	279.2330	0.36	111.3027	[23]
20	7.11	α or γ -Linolenic acid	ESI−	C_18_ H_30_ O_2_	N.D	277.2173	277.2173	0	69.9031, 59.0124	[21]
21	7.43	α or γ -Linolenic acid	ESI−	C_18_ H_30_ O_2_	N.D	277.2173	277.2173	0	69.9031, 59.0124	[21]
22	8.11	Arachidonic acid	ESI−	C_20_ H_32_ O_2_	N.D	303.2329	303.233	−0.33	287.0870, 259.2397	[21,23]
23	6.86	Eicosapentaenoic acid	ESI−	C_20_ H_30_ O_2_	N.D	301.2177	301.2173	0.33	273.5861, 257.2269	[21,24]
24	4.19	5 or 15-HEPE	ESI−	C_20_H_30_O_3_	N.D	317.2124	317.2122	0.63	255.2231, 115.0391	[24]
25	3.72	5 or 15-HEPE	ESI−	C_20_H_30_O_3_	N.D	317.2132	317.2122	3.15	255.2231	[24]
26	7.63	Docosahexaenoic acid	ESI−	C_22_ H_32_ O_2_	N.D	327.2331	327.233	0.31	283.2432, 269.2458	[25]
27	5.23	4,5-Epoxy-17R-HDHA	ESI+	C_25_H_34_O_4_	N.D	399.2519	399.253	−2.76	321.2201, 296.2936	[25]
28	12.14	6-bromo- Δ5-heptacosadienoic acid	ESI+	C_27_H_50_^79^Br0_2_^-^	N.D	485.3008, 487.2993	485.2989, 487.2989	−3.77, −0.82	405.3321, 112.5382, 57.0341	[22]
29	18.85	C18:3 Δ6, Δ9, Δ12/C19:1 Δ9 phosphatidylinositol	ESI+	C_46_H_81_O_13_P	N.D	873.5482	873.5488	−0.69	595.2531	[26]
30	15.73	C16: 1 Δ9/C16:0 phosphatidylglycerol	ESI+	C_38_H_73_O_1_0P	N.D	721.5011	721.5014	−0.42	663.4510, 193.1956	[27]
31	6.28	C18: 3 Δ9, Δ12, Δ15/C13:0 phosphatidylglycerol	ESI+	C_37_H_67_O_1_0P	N.D	703.4542	703.4545	0.21	671.4276, 643.4357	[27]
32	6.8	C20:1 Δ 11/C0:0 phosphatidic acid	ESI+	C_23_H_45_O_7_P	N.D	465.2977	465.2976	0.21	251.1775, 197.1311	[27,28]
33	10.89	C22: 3 Δ10, Δ13, Δ16/C22: 5 Δ7, Δ10, Δ13, Δ16, Δ19/C0: 0 diacylglycerol	ESI+	C_47_H_76_O_5_	N.D	721.5760	721.5765	0	316.5049, 313.1947, 173.1183	[29]
34	12.31	C18: 3 Δ9, Δ12, Δ15/C19: 1 Δ9/C20: 5 Δ5, Δ8, Δ 11, Δ14, Δ17 triacylglycerol	ESI+	C_60_H_98_O_6_	N.D	915.7436	915.7407	3.17	469.3639	[6]
35	12	C18: 3 Δ6, Δ9, Δ12/C22:4 Δ7, Δ10, Δ13, Δ16 phosphatidic acid	ESI+	C_43_H_71_O_8_P	N.D	747.4981	747.4959	2.94	732.1207, 101.112	[29]
36	11.69	C20: 5 Δ5, Δ8, Δ 11, Δ14, Δ17/C15: 0 phosphatidylcholine	ESI+	C_43_H_76_NO_8_P	N.D	766.5370	766.5381	−1.43	740.5090, 631.427	[29]
37	3.06	5-Androstenetriol	ESI+	C_19_H_30_O_3_	N.D	307.2272	307.2268	1.3	289.1795	[29]
38	9.88	C18: 1 Δ9/C12: 0 phosphatidic acid	ESI−	C_33_H_63_O_8_P	N.D	617.4211	617.4188	3.72	301.2803	[30,31]
39	13.62	C17: 0/C22: 5 Δ7, Δ10, Δ13, Δ16, Δ19/C0: 0 diacylglycerol	ESI−	C_42_H_72_O_5_	N.D	655.5309	655.5307	0.31	356.3249, 256.2157, 115.0359	[28]
40	6.66	C20: 3 Δ8, Δ11, Δ14/C20: 5 Δ5, Δ8, Δ11, Δ14, Δ17/C0: 0 diacylglycerol	ESI−	C_43_H_68_O_5_	N.D	663.5026	663.4994	4.82	375.2085, 112.9845	[27,29]
41	18.04	C22: 2 Δ13, Δ16/C22: 6 Δ4, Δ7, Δ10, Δ13, Δ16, Δ19/C0: 0 diacylglycerol	ESI−	C_47_H_76_O_5_	N.D	719.5636	719.562	2.22	664.5043, 440.8061	[29,30]
42	25.9	C16:0/C18: 2 Δ9, Δ12 diacylglyceryl-*N*,*N*,*N*-trimethyl homoserine (DGTS)	ESI−	C_44_H_81_NO_7_	N.D	734.5950	734.594	1.36	386.8379	[31]
43	11.22	C16: 1 Δ9/C20: 5 Δ5, Δ8, Δ11, Δ14, Δ17 phosphatidylglycerol	ESI−	C_42_H_71_O_10_P	N.D	765.4713	765.4712	0.13	529.2575, 253.2171	[30,32]
44	21.35	O-C18:0/C19:0 phosphatidylserine	ESI−	C_43_H_86_NO_9_P	N.D	790.5969	790.5968	0.13	283.5096, 73.2201	[27,32]
45	11.47	C16:0/C16:0 sulfoquinovosyl diacylglyceride (SQDG)	ESI−	C_41_H_78_O_12_S	N.D	793.5125	793.5141	−2.02	227.20132	[33]
46	1.58	Crocetin dialdehyde	ESI+	C_20_H_24_O_2_	N.D	297.1844	297.1849	−1.68	213.1372	[34,35]
47	5.42	*N*-butyl-*N*(2)-[ 2-hydroxy-2-phenylacetyl]-l-serinamide	ESI+	C_15_H_22_N_2_O_4_	N.D	295.1634	295.1652	−6.1	277.2147, 221.118	[6]
48	1.81	Formyl 2,4,6-decatrienoate	ESI+	C_11_H_16_O_2_	N.D	181.1217	181.1223	−3.31	163.111, 135.1164	[36]
49	1.29	Dipentyl phthalate	ESI+	C_18_H_26_O_4_	N.D	307.1889	307.1904	−4.88	163.111, 135.1164	[6]
50	3.98	Diisooctyl phtalate	ESI+	C_24_H_38_O_4_	N.D	413.2677	413.2671	1.45	381.2407, 363.2321	[6]
51	3.57	9,11 pentadecadienal	ESI+	C_15_H_26_O	N.D	223.2047	223.2056	−4.03	205.1954, 193.1005	[37]
52	10.26	*N*-stearoyl taurine	ESI+	C_20_H_41_NO_4_S	N.D	392.2864	392.2829	8.92	150.0262, 149.0229	[6]
53	1.07	(2,4)-*N*-isobutyl-6-phenyl-hexa-2,4-dienamide	ESI+	C_16_H_21_NO	N.D	244.1696	244.1696	0	226.1438, 184.0968	[6]
54	6.97	*N*-palmitoyl proline	ESI−	C_21_H_39_NO_3_	N.D	352.2859	352.2857	0.56	116.0705	[6]

*t***_R_** = Retention time; N.D = Not Determined; Ref. = Reference.

**Table 2:** Validation parameters of the proposed method.

Compounds	Retention Time (R*_t_*)	Linearity (*R*^2^)	Slope	LOQ (mg/mL)	LOD (mg/mL)
Fucoxanthin	8.15	0.9994	1.84 × 10^−5^	0.0000162	0.0000049
Astaxanthin	9.51	0.9998	0.00065	0.0001579	0.0000474
Zeaxanthin	10.52	0.9967	0.00015	0.0000019	0.0000006
Lutein	10.53	0.9991	0.01421	0.0029388	0.0008816

**Table 3:** The concentration of targeted metabolites in chloroform and acetone extracts of microalgal diatom *C. calcitrans.*

Extract	Metabolites	Concentration in Diatom in 100 mg of Extracts (µg/mg)
Chloroform	Fucoxanthin	1.2537 ± 0.0896
	Astaxanthin	0.3794 ± 0.0263
	Zeaxanthin	0.1503 ± 0.0001
	Lutein	0.1513 ± 0.0584
Acetone	Fucoxanthin	1.3031 ± 0.0521
	Astaxanthin	0.4346 ± 0.0438
	Zeaxanthin	0.1582 ± 0.0003
	Lutein	0.2414 ± 0.0325

## Supplementary Materials

The following are available online at https://www.mdpi.com/1660-3397/18/8/403/s1, Figure S1–S4: UHPLC-ESI-Orbitrap MS base peak chromatogram of the acetone, 70% ethanol, methanol and hexane extracts. Figure S5: The validation PLS models using permutation test for positive ionization (A: NO, B: DPPH) and negative ionisation (C: NO, D: DPPH) UHPLC-MS based metabolite profiling data of *C. calcitrans* whereas Figure S6: The PLS derived relationship between observed vs. predicted for positive ionization (A: NO, B: DPPH) and negative ionization (C: NO, D: DPPH) UHPLC-MS based metabolite profiling data of *C. calcitrans*. Table S1: Relative quantification of compounds in the extracts of *Chaetoceros calcitrans*.
