# Supplementary material for: Mass Spectrometry-Based Metabolomics Combined with Quantitative Analysis of the Microalgal Diatom (Chaetoceros calcitrans)
Source: Mar Drugs. 2020 Jul 30;18(8):403. doi: 10.3390/md18080403 (PMC7459737; doi:10.3390/md18080403)
Supplement: Supplementary file 1 [file marinedrugs-18-00403-s001.pdf]

Supplementary

Mass Spectrometry-Based Metabolomics Combined with Quantitative Analysis of the Microalgal Diatom (*Chaetoceros calcitrans*)

Awanis Azizan [1], M. Maulidiani [1,2], Rudiyanto, R. [3], Khozirah Shaari [1,4], Intan Safinar Ismail [1,4], Norio Nagao [5] and Faridah Abas [1,6]*

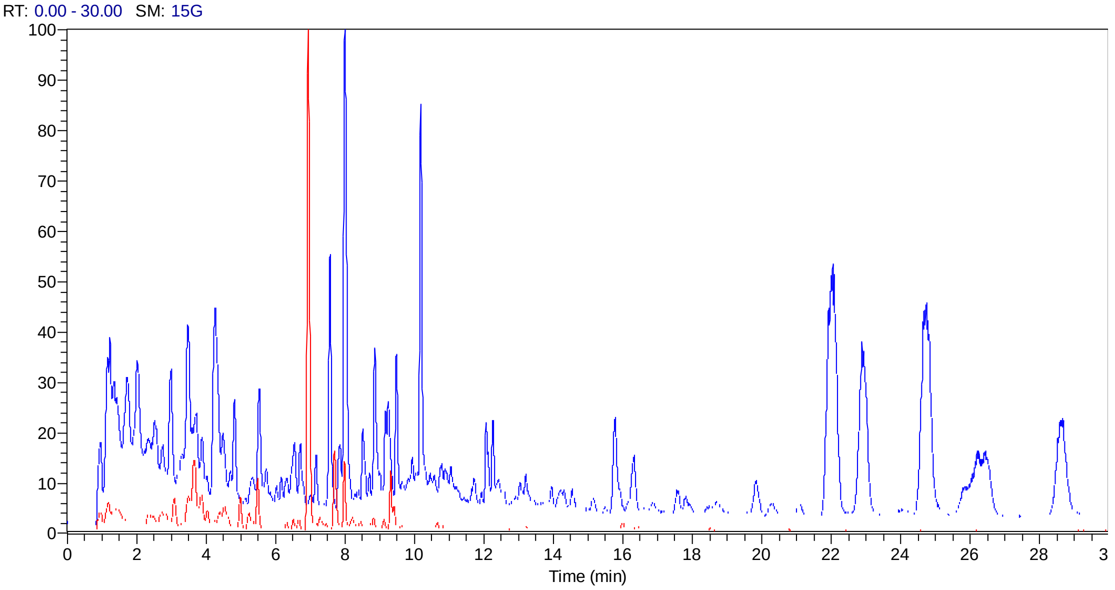

Figure S1. UHPLC-ESI-Orbitrap MS base peak chromatogram of the acetone extract. Retention time (0-30 minutes) obtain from microalga diatom *C. calcitrans*. Blue color represents the chromatogram acquired in positive ion mode whereas red color represents the negative ion mode.

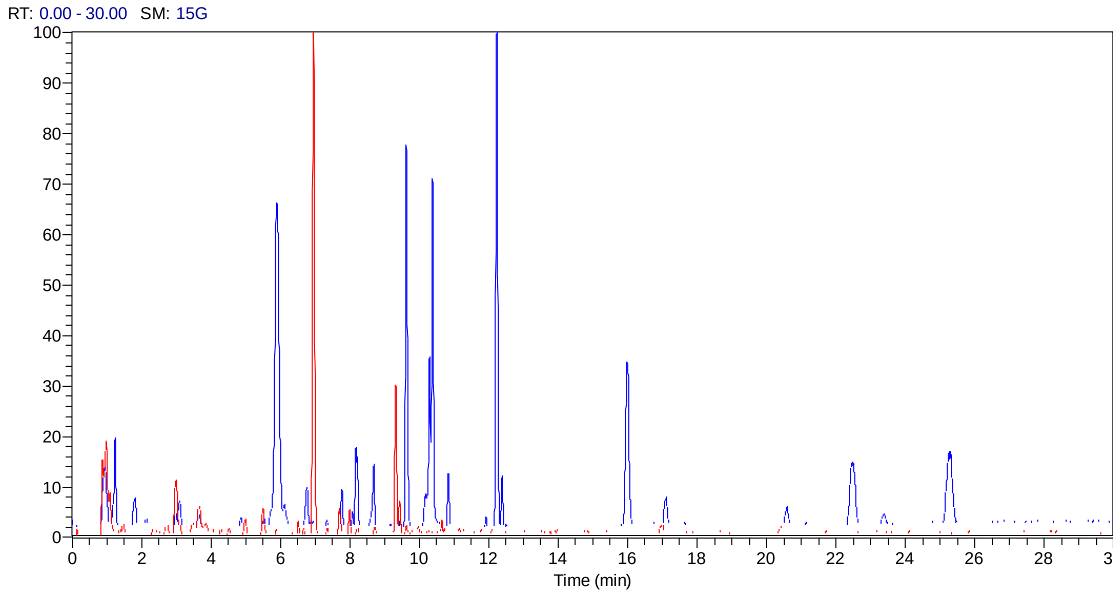

Figure S2. UHPLC-ESI-Orbitrap MS base peak chromatogram of the70% ethanol extract. Retention time (0-30 minutes) obtain from microalga diatom *C. calcitrans*. Blue color represents the chromatogram acquired in positive ion mode whereas red color represents the negative ion mode.

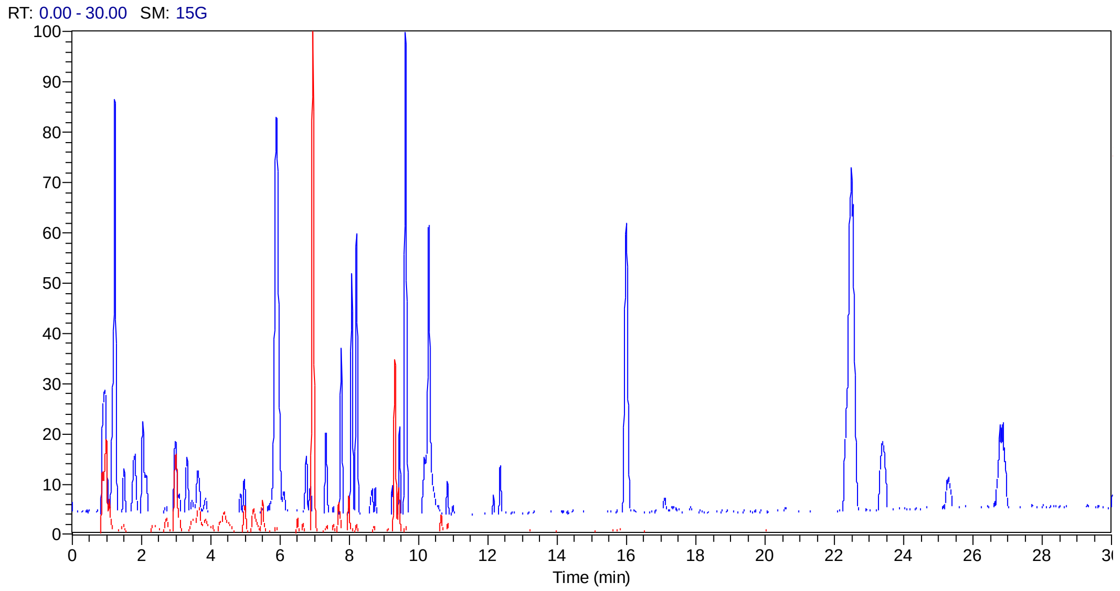

Figure S3. UHPLC-ESI-Orbitrap MS base peak chromatogram of the methanol extract. Retention time (0-30 minutes) obtain from microalga diatom *C. calcitrans*. Blue color represents the chromatogram acquired in positive ion mode whereas red color represents the negative ion mode.

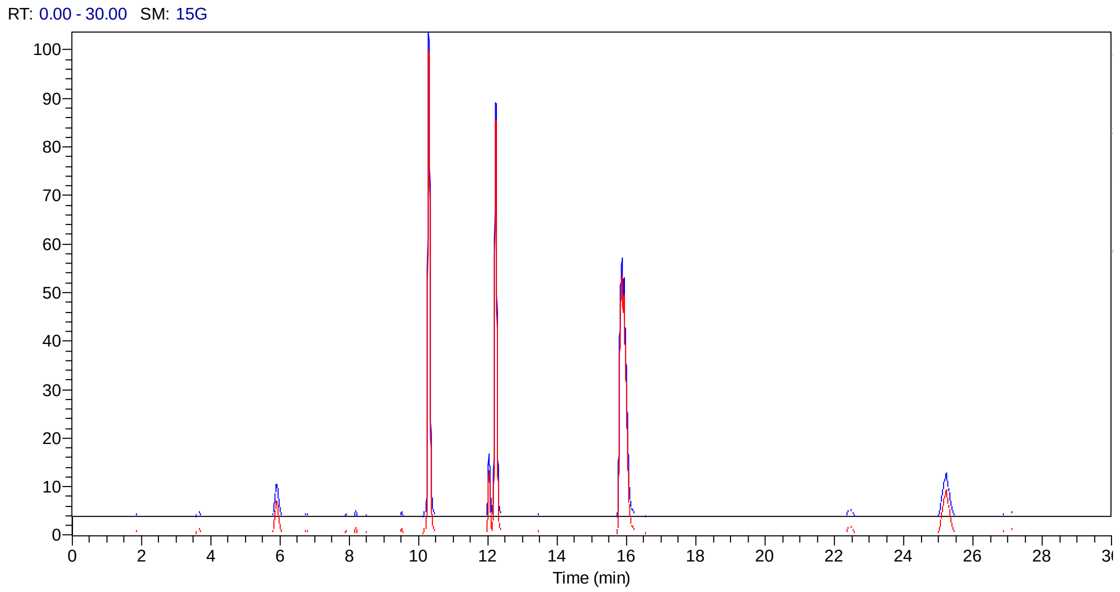

Figure S4. UHPLC-ESI-Orbitrap MS base peak chromatogram of the hexane extract. Retention time (0-30 minutes) obtain from microalga diatom *C. calcitrans*. Blue color represents the chromatogram acquired in positive ion mode whereas red color represents the negative ion mode.

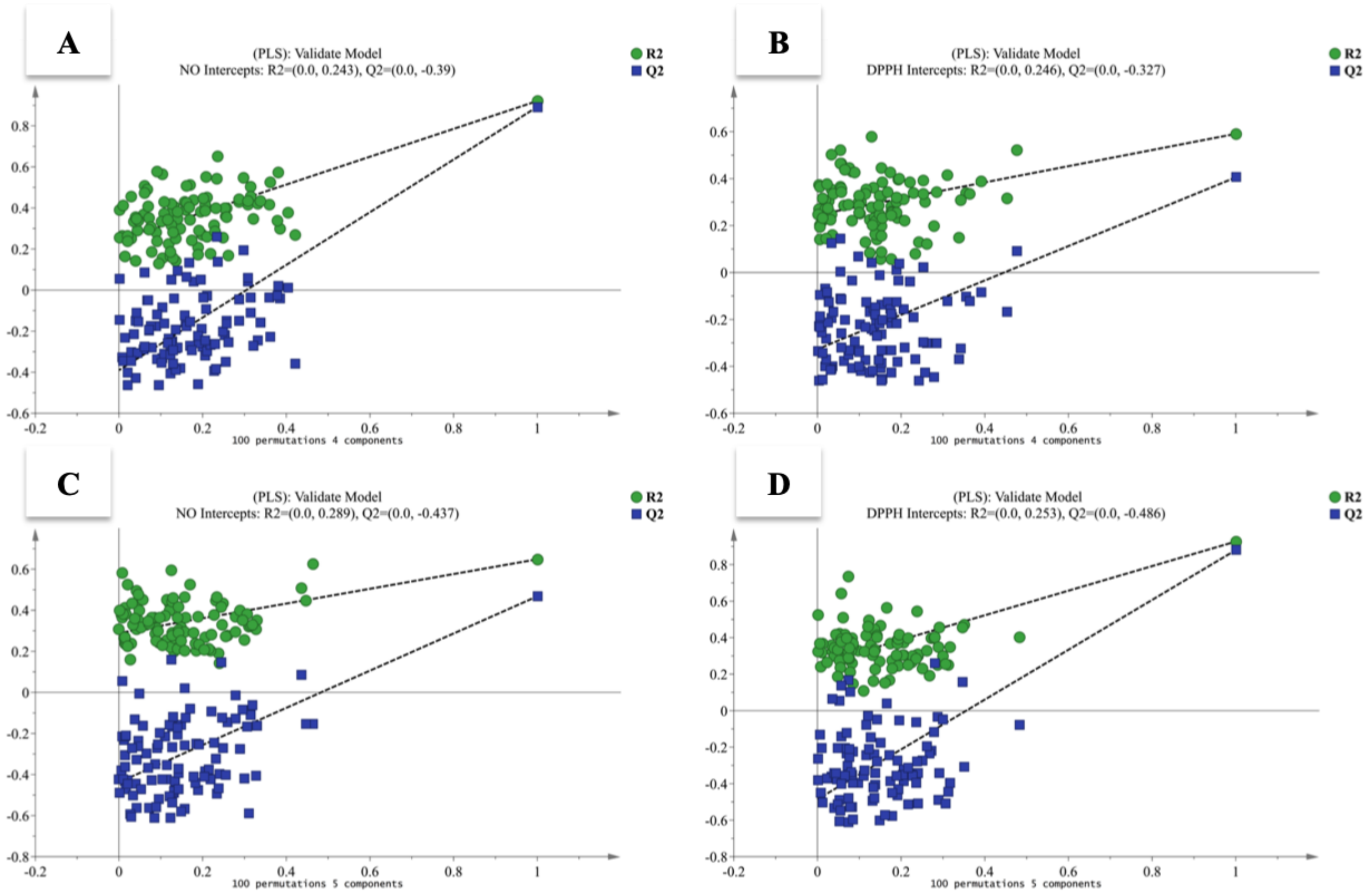

Figure S5. Validation PLS models using permutation test for positive ionization (A: NO, B: DPPH) and negative ionisation (C: NO, D: DPPH) UHPLC-MS based metabolite profiling data of *C. calcitrans* .

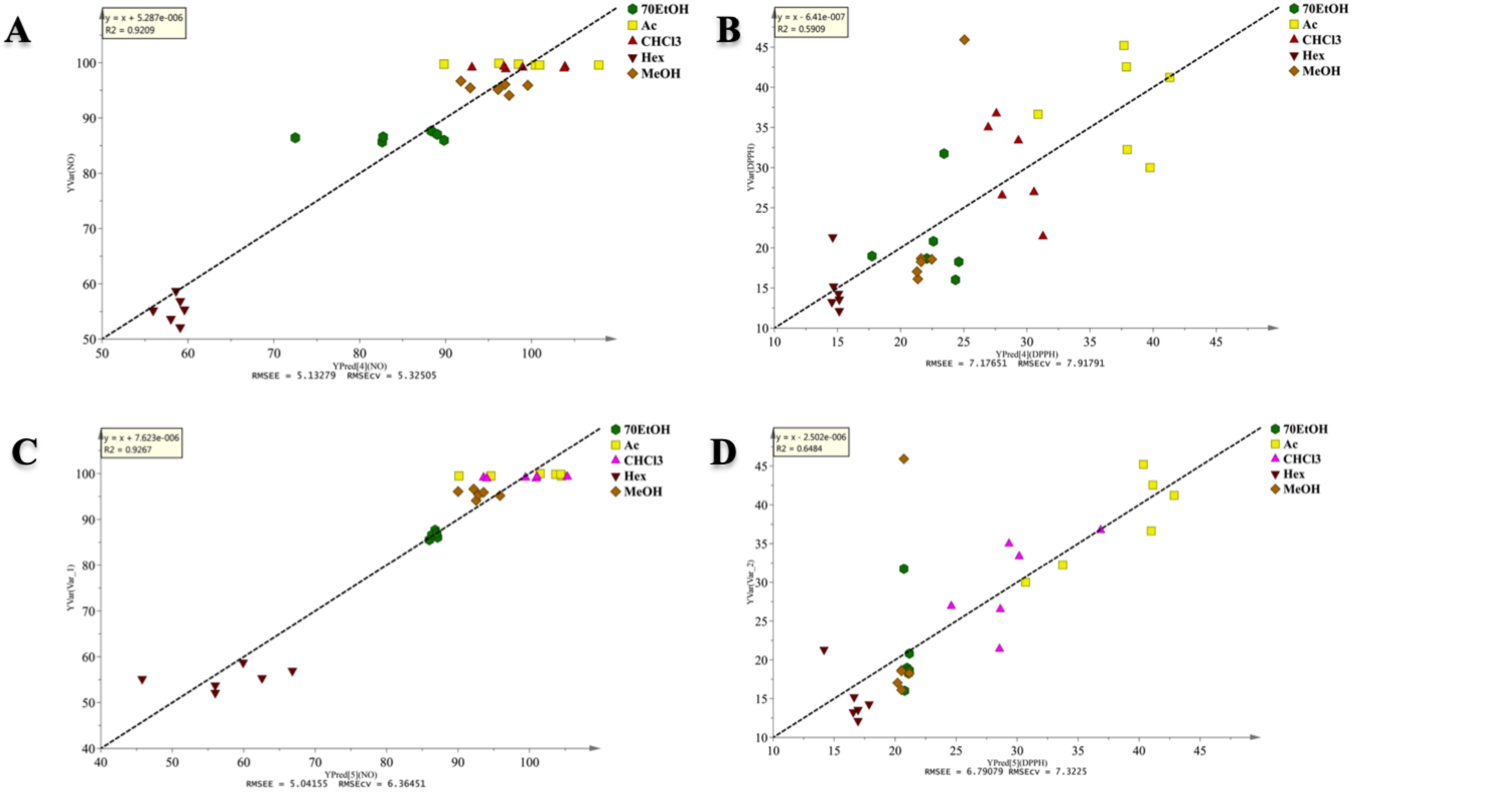

Figure S6. PLS derived relationship between observed vs predicted for positive ionization (A: NO, B: DPPH) and negative ionisation (C: NO, D: DPPH) UHPLC-MS based metabolite profiling data of *C. calcitrans* .

Table 1S. Relative quantification of compounds in the extracts of *Chaetoceros calcitrans*.

Var ID (mzmed)	Var ID (metabolites)	VIP	*P value*[b]									
			A vs C	A vs H	A vs M	A vs 7E	C vs H	C vs M	C vs 7E	H vs M	H vs 7E	M vs 7E
**Positive ion mode**												
658.423	Fucoxanthin	2.840	0.898	0.000	0.000	0.000	0.000	0.000	0.000	0.105	0.001	0.218
765.483	C20: 5 Δ5, Δ8, Δ 11, Δ14, Δ17/ C15:0 phosphatidylcholine (PC)	1.711	0.000	0.000	0.117	0.000	0.465	0.000	0.000	0.000	0.000	0.000
273.222	9E,11Z pentadecadienal	1.433	0.976	0.000	0.000	0.000	0.000	0.000	0.000	0.520	0.120	0.884
618.425	C18: 1 Δ9 / C12: 0 phosphatidic acid (PA)	1.355	0.198	0.000	0.000	0.000	0.000	0.000	0.000	0.999	0.992	1.000
616.413	Fucoxanthinol	1.120	0.003	0.000	0.000	0.000	0.000	0.000	0.000	0.464	0.893	0.935
608.26	Chlorophyll c2	1.077	0.000	0.120	0.000	0.000	0.000	0.000	0.000	0.078	0.080	1.000
568.409	Lutein	1.018	0.761	0.000	0.000	0.000	0.000	0.000	0.000	1.000	1.000	1.000
391.286	N-stearoyl taurine	1.009	0.381	0.000	0.000	0.000	0.000	0.000	0.000	0.479	0.063	0.767
600.399	Neoxanthin	1.001	0.001	0.000	0.000	0.000	0.000	0.000	0.000	1.000	1.000	1.000
**Negative ion mode**												
318.215	5 or 15-HEPE	2.065	0.193	0.000	0.000	0.000	0.000	0.000	0.000	0.466	0.147	0.948
328.236	Docosahexanoic acid	2.019	0.636	0.000	0.000	0.000	0.000	0.000	0.000	0.002	0.000	0.927
318.215	5 or 15-HEPE	1.771	0.101	0.000	0.000	0.000	0.000	0.000	0.000	0.732	0.874	0.218
254.22	3-Hexadecenoic acid	1.356	0.998	0.000	0.000	0.000	0.000	0.000	0.000	0.293	0.979	0.608
302.221	Eicosapentaenoic acid	1.190	0.000	0.000	0.000	0.000	0.962	0.953	0.953	1.000	1.000	1.000
304.236	Arachidonic acid	1.118	0.622	0.000	0.000	0.000	0.000	0.000	0.000	0.007	0.001	0.960

P values were results of Tukey-HSD pairwise multiple-comparison tests using SPSS 16.0. Significant level: $P > 0.050$, not significant; $0.050 \geq P > 0.010$, significant*; $0.010 \geq P > 0.001$, very significant**; and $0.001 \geq P$, highly significant***. Letters indicate the three extraction solvents of *Chaetoceros calcitrans*: (A) Acetone, (C) $CHCl_3$, (H) Hexane, (M) Methanol and (7E) 70% Ethanol.
